# Prediction of obstructive sleep apnea: comparative performance of three screening instruments on the apnea-hypopnea index and the oxygen desaturation index

**DOI:** 10.1007/s11325-020-02219-6

**Published:** 2020-10-24

**Authors:** Christianne C. A. F. M. Veugen, Emma M. Teunissen, Leontine A. S. den Otter, Martijn P. Kos, Robert J. Stokroos, Marcel P. Copper

**Affiliations:** 1 Department of Otorhinolaryngology Head and Neck surgery, Sint Antonius Hospital, Koekoekslaan 1, 3435 CM Nieuwegein, The Netherlands; 2 Department of Otorhinolaryngology Head and Neck surgery, Universitair Medisch Centrum Utrecht, Heidelberglaan 100, 3584 CX Utrecht, The Netherlands; 3 Department of Otorhinolaryngology Head and Neck surgery, Radboud Universitair Medisch Centrum, Geert Groteplein Zuid 10, 6525 GA Nijmegen, The Netherlands; 4 Faculty of Medicine, Universitair Medisch Centrum Groningen, Hanzeplein 1, 9713 GZ Groningen, The Netherlands; 5 Ruysdael Sleepclinic, Ruysdaelstraat 49 A1-D, 1071 XA Amsterdam, The Netherlands

**Keywords:** Obstructive sleep apnea, Polysomnography, Screening, NoSAS score, STOP-Bang questionnaire, ESS

## Abstract

**Purpose:**

To evaluate the performance of the NoSAS (neck, obesity, snoring, age, sex) score, the STOP-Bang (snoring, tiredness, observed apneas, blood pressure, body mass index, age, neck circumference, gender) questionnaire, and the Epworth sleepiness score (ESS) as a screening tool for obstructive sleep apnea (OSA) severity based on the apnea-hypopnea index (AHI) and the oxygen desaturation index (ODI).

**Methods:**

Data from 235 patients who were monitored by ambulant polysomnography (PSG) were retrospectively analyzed. OSA severity was classified based on the AHI; similar classification categories were made based on the ODI. Discrimination was assessed by the area under the curve (AUC), while predictive parameters were calculated by four-grid contingency tables.

**Results:**

The NoSAS score and the STOP-Bang questionnaire were both equally adequate screening tools for the AHI and the ODI with AUC ranging from 0.695 to 0.767 and 0.684 to 0.767, respectively. Both questionnaires perform better when used as a continuous variable. The ESS did not show adequate discrimination for screening for OSA (AUC ranging from 0.450 to 0.525). Male gender, age, and BMI proved to be the strongest individual predictors in this cohort.

**Conclusion:**

This is the first study to evaluate the predictive performance of three different screening instruments with respect to both the AHI and the ODI. This is important, due to increasing evidence that the ODI may have a higher reproducibility in the clinical setting. The NoSAS score and the STOP-Bang questionnaire proved to be equally adequate to predict OSA severity based on both the AHI and the ODI.

**Electronic supplementary material:**

The online version of this article (10.1007/s11325-020-02219-6) contains supplementary material, which is available to authorized users.

## Introduction

Obstructive sleep apnea (OSA) is a sleep-related breathing disorder characterized by repetitive partial or complete upper airway obstruction which often results in decreased arterial oxygen saturation and arousal from sleep [1]. OSA severity is commonly classified based on the apnea-hypopnea index (AHI) [2]. OSA has been associated with cardiovascular and metabolic consequences and is also linked with increased overall mortality [3]. Currently, overnight polysomnography (PSG) is the gold standard for diagnosing the presence and severity of OSA. However, its high expense, relative inaccessibility, and time consumption can delay or impede the diagnosis and treatment of patients with OSA, mainly in areas with limited healthcare resources [4]. Additionally, the increasing number of patients suspected of having OSA and the lack of structured patient interviews contribute to the growing number of patients being referred to sleep clinics [5]. Therefore, simple screening instruments for identifying patients at high risk for OSA have become increasingly important.

Several instruments have been developed over the years including the STOP-Bang questionnaire [6, 7] and the NoSAS score [8]. The STOP-Bang questionnaire shows a high sensitivity and negative predictive value, and therefore is a suitable instrument to rule out patients at risk for OSA [9–12]. However, it has a low to moderate specificity and it is possible that this will yield a high false-positive rate. Low specificity may result in unnecessary referral to sleep clinics for polysomnography [6, 7]. The NoSAS score has been validated in multiple patient cohorts, and opinions concerning superiority over the STOP-Bang questionnaire differ [8, 10, 13–15]. The original validation of the NoSAS score by Marti-Soler et al. describes higher specificity and positive predictive values in comparison with the STOP-Bang questionnaire, while maintaining a moderate to high sensitivity and negative predictive value, therefore allowing to rule out clinically significant OSA and simultaneously reducing the number of unnecessary nocturnal recordings as well as the number of missed diagnosis [8]. The Epworth sleepiness scale (ESS), which was originally designed to assess the extent of daytime sleepiness, has also been suggested as a screening tool for identifying patients at high risk for OSA [16]. However, multiple authors have found the ESS to be inferior to other screening tools for identifying patients at high risk for OSA [11, 12, 17, 18].

The present study reviewed and analyzed a cohort of 235 patients who underwent PSG, using in each case all three instruments: the STOP-Bang questionnaire [6], the NoSAS score [8], and the ESS [16]. Our main objectives were to evaluate the predictive and discriminative performance of the different screening instruments and compare the diagnostic effectiveness of the different methods. Additionally, we aimed to determine which variables independently were the strongest predictors in this cohort.

Recently, it has been suggested that the AHI is susceptible to variability in the clinical setting and that there is a need for an alternative parameter to indicate OSA severity [3, 19–21]. An important disadvantage regarding the AHI is that the morphology and duration of the apneas are not taken into account. Longer, deeper apneas might be more significant than shorter, shallow ones [22]. Significant differences in the severity of OSA have been described between patients with a similar AHI [22]. Nocturnal oxygen desaturations are the result of apneas and are important in the pathogenesis and development of complications of OSA [23]. The arterial oxygen desaturation index (ODI) has therefore been proposed as an alternative for the AHI in grading PSG data and classifying OSA severity [23–26]. The ODI might be more relevant due to the higher reproducibility in the clinical setting [3, 19–21]. Furthermore, there is evidence that the ODI is independently associated with prevalent risk factors like hypertension, whereas the AHI is not [19]. Therefore, in the present study, the discriminatory ability of the screening instruments will be evaluated by criteria based on the AHI as well as on the ODI.

## Methods

### Study design

Data from 235 patients who were monitored by ambulant PSG were retrospectively analyzed. Patient inclusion criteria were patients aging 18 years of age or older, completed clinical data, and completed STOP-Bang questionnaire and NoSAS score. Patient exclusion criteria were previously diagnosed OSA, use of portable sleep studies or respiratory polygraphy, incomplete clinical data, and technically inadequate PSG. In the outpatient clinic, the following clinical parameters were collected for all patients: gender, age, height, weight, body mass index (BMI), neck circumference (NC), self-reported complaints (snoring, daytime sleepiness, and apnea), and self-reported comorbidities (cardiovascular history, hypertension, pulmonary history). The ESS was completed. The clinical parameters were used to calculate the NoSAS score and the STOP-Bang questionnaire.

### Screening instruments (supplementary material)

The STOP-Bang questionnaire consists of four questions used in the STOP questionnaire—snoring, tiredness, observed apneas, and hypertension—plus four demographic queries—BMI > 35 kg/m^2^, age > 50 years old, neck circumference > 40 cm, and male gender. For each question, answering ‘yes’ scores 1, a ‘no’ scores 0. With a total range of 0–8, a total score of ≥ 3 points is considered as a high probability for OSA [6]. The NoSAS score is a 5-item questionnaire which includes neck circumference, obesity, snoring, age, and gender. With a range of 0–17, NoSAS scores 4 points for neck circumference ≥ 40 cm, 3 points for BMI 25–30 kg/m^2^, 5 points for BMI ≥ 30 kg/m^2^, 2 points for snoring, 4 points for age > 55 years old, and 2 points for male gender. The total score of ≥ 8 points is considered as a high probability for OSA [8]. The ESS consists of 8 situations, allowing the patients to assess their degree of dozing off or falling asleep in a particular scene during the day, 0 for no dozing, and 1, 2, and 3 for slight, moderate, and high chance of dozing. A total score of ≥ 10 points is considered as excessive daytime sleepiness [16].

### Sleep study, scoring, and diagnosis

All patients underwent a full-night PSG at home. PSG included electroencephalography, electrooculography, surface electromyography, nasal airflow and air temperature, thoracoabdominal movements, pulse oximetry, body position, and snoring sounds. Breathing was recorded with nasal pressure and temperature sensors. Scoring of the electronic raw data was performed manually, following the recommendations of the American Academy of Sleep Medicine [2]. Apnea was defined as a decrease of at least 90% of airflow from baseline for > 10 s. Hypopnea was defined as a decrease of at least 30% of airflow from baseline for > 10 s, associated with either an arousal or ≥ 3% arterial oxygen saturation decrease. The mean number of apneas and hypopneas per hour of sleep (AHI) was calculated. The ODI was defined as the mean number of arterial oxygen desaturations ≥ 3% per hour. The severity of OSA was categorized both according to the AHI and to the ODI. By using the AHI, patients were classified as mild (5 ≤ AHI < 15 events/h), moderate (15 ≤ AHI < 30 events/h), or severe (AHI ≥ 30 events/h) according to the 2012 American Academy of Sleep Medicine criteria [2]. For classification according to the ODI, patients were divided into similar groups: mild (5 ≤ ODI < 15 events/h), moderate (15 ≤ ODI < 30 events/h), and severe (ODI ≥ 30 events/h) [27]. Other PSG parameters collected included the apnea index (AI), the AHI in supine position, the AHI in non-supine position, minimal arterial oxygen saturation (minimal SpO_2_), baseline arterial oxygen saturation (baseline SpO_2_), average arterial oxygen saturation (average SpO_2_), and percentage of sleep time with arterial oxygen saturation time below 90% (SpO_2_ time < 90%).

### Statistical analysis

The statistical analysis was performed by using Statistical Package for Social Studies (IBM SPSS Statistics version 24 for Windows, New York, NY, USA). Continuous data are presented as means with standard deviations. Categorical variables are presented as frequencies with percentages. Comparisons between groups were performed using Chi-square tests for categorical variables, unpaired Student’s *t* test, and univariate analysis of variance (ANOVA) for continuous variables. Discrimination, the ability of a screening tool to distinguish between patients with and without different outcomes, was estimated from the area under the curve (AUC) obtained by receiver operator characteristic (ROC) curves, which may range from 0.5 (no discrimination) to 1.0 (perfect discrimination) [28]. The AUCs were compared using the algorithm previously described by Hanley et al. [29]. Additionally, sensitivity, specificity, positive predictive value (PPV), and negative predictive value (NPV) were calculated for different AHI and ODI cutoffs using four-grid contingency tables, all estimates are reported with their respective 95% confidence interval (CI). The association between various individual demographic and clinical variables and the presence and degree of OSA was established by using a multivariate logistic regression model (backward stepwise selection, *p* < 0.05). A two-tailed *p* value < 0.05 was considered statistically significant.

## Results

### Baseline characteristics

A total of 201 patients met our inclusion criteria; baseline characteristics are mentioned in Table 1. A total of 148 (73.6%) patients were male, aged 50.0 ± 12.6 years, with a mean BMI of 28.0 ± 4.8 kg/m^2^. Based on the AHI, OSA was present in 159 (79.1%) of the patients; 66 (41.5%) with mild OSA, 45 (28.3%) with moderate OSA, and 48 (30.2%) with severe OSA. Male gender, age, BMI, neck circumference, cardiovascular history, hypertension, snoring, and apneas were all significantly higher in the OSA groups than in the no OSA group. A post hoc Bonferroni test showed a statistically significant difference between no OSA and moderate/severe OSA for male gender (*p* = 0.008; *p* = 0.001), age (*p* = 0.002; *p* = 0.013), and BMI (*p* = 0.045; *p* < 0.001). BMI was also significantly different between mild/moderate OSA and severe OSA (*p* < 0.001; *p* = 0.030). Neck circumference (*p* = 0.043; *p* = 0.032), cardiovascular history (*p* = 0.006; *p* = 0.040), and hypertension (*p* = 0.004; *p* = 0.002) all showed a statistically significant difference between no/mild OSA and severe OSA. The ESS did not differ significantly between OSA groups (*p* = 0.667; *p* = 0.616). A total of 54.5%, 75.6%, and 85.4% of the patients in the mild, moderate, and severe OSA group, respectively, were classified as high risk of OSA according to the NoSAS score (cutoff ≥ 8; *p* < 0.001). A total of 97%, 100%, and 100% in the mild, moderate, and severe OSA group, respectively, were classified as high risk of OSA according to the STOP-Bang questionnaire (cutoff ≥ 3; *p* < 0.001). Polysomnography results (AHI, ODI ≥ 3%, minimal SpO_2_, average SpO_2_, and SpO_2_ time < 90%) were all significantly different between the OSA and no OSA groups (*p* < 0.001; *p* < 0.001; *p* < 0.001; *p* < 0.001; *p* = 0.001). Notable is the percentage of patients with positional sleep apnea which was also statistically significant between the groups (*p* < 0.001). A post hoc Bonferroni test shows that the difference was significant between no OSA and all OSA severity groups (*p* < 0.001) and mild OSA and severe OSA (*p* = 0.05).

**Table 1:** Baseline characteristics

	All patient (*n* 201)	No OSA (AHI ≤ 5) (*n* 42)	Mild OSA (5 ≤ AHI < 15) (*n* 66)	Moderate OSA (15 ≤ AHI < 30)(*n* 45)	Severe OSA (AHI ≥ 30) (*n* 48)	*p* value
Male patients	148 (73.6%)	22 (52.4%)	47 (71.2%)	37 (82.2%)	42 (87.5%)	*0.001*
Age (year)	50.0 ± 12.6	44.3 ± 11.0	49.3 ± 11.8	54.0 ± 11.0	52.3 ± 13.7	*0.002*
BMI (kg/m^2^)	28.0 ± 4.8	25.9 ± 3.4	26.7 ± 4.2	28.5 ± 4.0	31.1 ± 5.8	*< 0.001*
NC > 40 (cm)	100 (49.8%)	17 (40.5%)	28 (42.4%)	22 (48.9%)	33 (68.8%)	*0.020*
Cardiovasc. His.	59 (29.4%)	6 (14.3%)	15 (22.7%)	16 (35.6%)	22 (45.8%)	*0.004*
Hypertension	46 (22.9%)	5 (11.9%)	9 (13.6%)	12 (26.7%)	20 (41.7%)	*0.001*
Pulm. His.	7 (3.5%)	3 (7.1%)	1 (1.5%)	0 (0%)	3 (6.3%)	0.813^a^
Snoring	190 (94.5%)	38 (90.5%)	61 (92.4%)	43 (95.6%)	45 (100%)	*0.033*^a^
Sleepiness	166 (82.6%)	38 (90.5%)	50 (75.8%)	37 (82.2%)	41 (85.4%)	0.238
Apneas	148 (73.6%)	27 (64.3%)	43 (65.2%)	36 (80.0%)	42 (87.5%)	*0.018*
ESS^b^	5.8 ± 3.6	6.1 ± 3.9	5.4 ± 3.6	5.6 ± 3.4	6.1 ± 3.6	0.667
ESS ≥ 10^b^	35 (17.4%)	9 (21.4%)	12 (19.4%)	5 (11.4%)	9 (19.6%)	0.616
NoSAS	9.5 ± 4.0	7.3 ± 3.9	8.6 ± 3.5	10.3 ± 3.6	12.0 ± 3.5	*< 0.001*
NoSAS ≥ 8	130 (64.7%)	19 (45.2%)	36 (54.5%)	34 (75.6%)	41 (85.4%)	*< 0.001*
Stop-Bang	4.6 ± 1.4	3.8 ± 1.4	4.2 ± 1.2	4.8 ± 1.2)	5.5 ± 1.3	*< 0.001*
Stop-Bang ≥ 3	192 (95.5%)	35 (83.3%)	64 (97%)	45 (100%)	48 (100%)	*< 0.001*^a^
AHI (e/h)	20.5 ± 18.8	3.2 ± 1.2	9.5 ± 3.0	22.2 ± 4.1	49.1 ± 18.8	*< 0.001*
ODI > 3% (e/h)	17.8 ± 17.3)	2.7 ± 1.4)	7.9 ± 3.3)	18.7 ± 5.2)	43.6 ± 14.5)	*< 0.001*
Positional OSA	109 (54.2%)	0 (0%)	53 (80.3%)	30 (66.7%)	26 (54.2%)	*< 0.001*
Min SpO_2_ (%)	84.8 ± 7.3	89.5 ± 3.4	87.1 ± 5.2	84.9 ± 3.9	77.6 ± 9.4	*< 0.001*
Average SpO_2_ (%)	94.1 ± 2.0	95.1 ± 1.6	94.3 ± 1.9	93.9 ± 1.6	93.2 ± 2.2	*< 0.001*
SpO_2_ time < 90% (%)	6.9 ± 14.8	3.0 ± 8.6	5.8 ± 17.2	4.5 ± 11.9	14.0 ± 15.9	*0.001*

Data are presented as mean ± standard deviation or number and percentage (%). Chi-square tests for categorical variables and ANOVA tests for continuous variables

*AHI* apnea-hypopnea index, *BMI* body mass index, *Cardiovasc. His.* cardiovascular history, *NC* neck circumference, *ODI* oxygen desaturation index, *Pulm. His.* pulmonary history

Italics is statistically significant

^a^Mann-Whitney *U* test

^b^Seven missing patients

### Performance of instruments

**Fig. 1:**
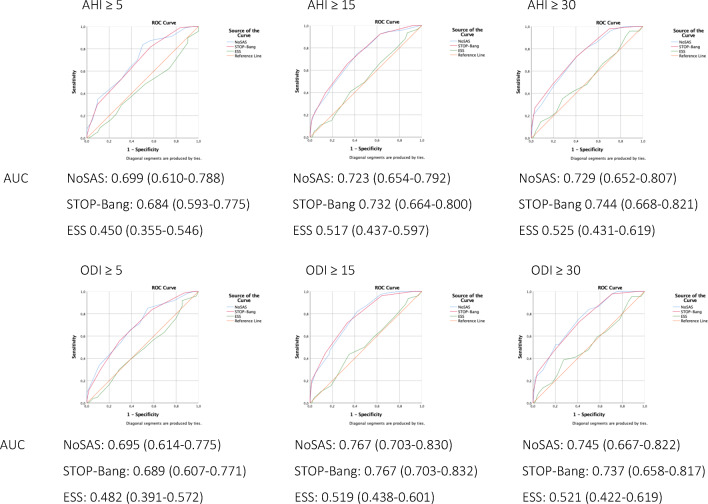
Discriminatory ability reported as area under the curve (AUC) (95% CI). The NoSAS score, the STOP-Bang questionnaire, and the ESS are presented as continuous variables. OSA severity is classified based on AHI ≥ 5 (any OSA), AHI ≥ 15 (moderate to severe OSA), and AHI ≥ 30 (severe OSA). The ODI ≥ 3% is subdivided into ODI ≥ 5, ODI ≥ 15, and ODI ≥ 30. The NoSAS score performed similar when compared with the STOP-Bang questionnaire on all cutoff points (all comparisons with *p* value > 0.05). The ESS presented lower discrimination than presented by the NoSAS score and the STOP-Bang questionnaire on all cutoff points (all comparisons with *p* value < 0.05)

The predictive performance of the different screening instruments as categorical variable is shown in Table 2. For screening on different cut-off points of AHI and ODI severity, the sensitivity of the NoSAS score varies from 0.70 to 0.92 (AHI > 5 and AHI > 15, respectively). The specificity varies from 0.37 to 0.55 (AHI > 15 and AHI > 5, respectively). The STOP-Bang questionnaire showed the highest sensitivity varying from 0.99 to 1.00. However, the specificity was lower varying from 0.06 to 0.17. The highest specificity was obtained by the ESS, varying from 0.79 to 0.83, with a low sensitivity varying from 0.15 to 0.19. Figure 1 shows the ROC curves and the corresponding AUC of the three screening instruments on different levels of AHI and ODI severity. The screening instruments are presented as continuous variables. The ESS did not show adequate discrimination for screening for AHI and ODI with an AUC ranging from 0.450 to 0.525. The NoSAS score and the STOP-Bang questionnaire were both equally adequate screening tools for the AHI and the ODI with AUC ranging from 0.695 to 0.767 and 0.684 to 0.767, respectively (all comparisons with *p* value > 0.05). The discriminatory ability of the NoSAS score and the STOP-Bang questionnaire was similar in relation to both the AHI and the ODI (all comparisons with *p* value > 0.05). When used as categorical variable, the AUC of the NoSAS score ranged from 0.620 to 0.684 (cutoff ≥ 8), the AUC of the STOP-Bang questionnaire ranged from 0.529 to 0.577 (cutoff ≥ 3) (Table 2). Both instruments performed better when used as continuous variable than as categorical variable. However, only for the STOP-Bang questionnaire, this difference proved to be significant (all comparisons except AHI ≥ 5 with *p* value < 0.05).

**Table 2:** Performance of the NoSAS score, the STOP-Bang questionnaire, and the ESS. The screening instruments are presented as categorical variables (NoSAS ≥ 8, STOP-Bang ≥ 3, ESS ≥ 10)

		AUC (95% CI)	Sensitivity (95% CI)	Specificity (95% CI)	PPV (95% CI)	NPV (95% CI)
AHI ≥ 5 e/h	NoSAS ≥ 8	0.623 (0.525–0.720)	0.70 (0.62–0.76)	0.55 (0.40–0.69)	0.85 (0.78–0.90)	0.32 (0.23–0.44)
	STOP-Bang ≥ 3	0.577 (0.473–0.681)	0.99 (0.96–1.00)	0.17 (0.08–0.31)	0.82 (0.76–0.87)	0.78 (0.45–0.94)
	ESS ≥ 10	0.478 (0.378–0.579)	0.16 (0.11–0.23)	0.79 (0.64–0.88)	0.74 (0.58–0.86)	0.2 (0.15–0.27)
AHI ≥ 15 e/h	NoSAS ≥ 8	0.649 (0.573–0.725)	0.92 (0.85–0.96)	0.37 (0.29–0.46)	0.56 (0.48–0.63)	0.85 (0.72–0.93)
	STOP-Bang ≥ 3	0.542 (0.462–0.621)	1.00 (0.96–1.00)	0.08 (0.04–0.15)	0.48 (0.41–0.55)	1.00 (0.70–1.00)
	ESS ≥ 10	0.477 (0.395–0.558)	0.15 (0.09–0.24)	0.81 (0.72–0.97)	0.4 (0.26–0.56)	0.52 (0.45–0.6)
AHI ≥ 30 e/h	NoSAS ≥ 8	0.636 (0.552–0.720)	0.85 (0.73–0.93)	0.42 (0.34–0.5)	0.32 (0.24–0.4)	0.9 (0.81–0.95)
	STOP-Bang ≥ 3	0.529 (0.438–0.620)	1.00 (0.93–1.00)	0.06 (0.03–0.11)	0.25 (0.19–0.32)	1.00 (0.70–1.00)
	ESS ≥ 10	0.510 (0.414–0.606)	0.19 (0.1–0.32)	0.83 (0.76–0.88)	0.26 (0.14–0.42)	0.77 (0.7–0.82)
ODI ≥ 5 e/h	NoSAS ≥ 8	0.620 (0.531–0.709)	0.71 (0.63–0.78)	0.53 (0.4–0.65)	0.80 (0.72–0.86)	0.41 (0.30–0.52)
	STOP-Bang ≥ 3	0.557 (0.464–0.650)	0.99 (0.95–1.00)	0.13 (0.06–0.24)	0.75 (0.68–0.81)	0.78 (0.45–0.94)
	ESS ≥ 10	0.484 (0.392–0.575)	0.16 (0.11–0.23)	0.80 (0.68–0.88)	0.69 (0.52–0.81)	0.27 (0.2–0.34)
ODI ≥ 15 e/h	NoSAS ≥ 8	0.684 (0.610–0.757)	0.87 (0.78–0.93)	0.50 (0.41–0.58)	0.52 (0.44–0.61)	0.86 (0.76–0.92)
	STOP-Bang ≥ 3	0.537 (0.456–0.617)	1.00 (0.95–1.00)	0.07 (0.04–0.13)	0.41 (0.34–0.48)	1.00 (0.7–1.00)
	ESS ≥ 10	0.483 (0.400–0.567)	0.15 (0.09–0.25)	0.81 (0.74–0.87)	0.34 (0.21–0.51)	0.60 (0.53–0.67)
ODI ≥ 30 e/h	NoSAS ≥ 8	0.639 (0.553–0.724)	0.86 (0.73–0.94)	0.41 (0.34–0.49)	0.29 (0.22–0.38)	0.92 (0.83–0.96)
	STOP-Bang ≥ 3	0.529 (0.435–0.622)	1.00 (0.92–1.00)	0.06 (0.03–0.11)	0.23 (0.18–0.29)	1.00 (0.70–1.00)
	ESS ≥ 10	0.506 (0.407–0.606)	0.18 (0.1–0.32)	0.83 (0.76–0.88)	0.23 (0.12–0.39)	0.78 (0.71–0.84)

*AHI* apnea-hypopnea index, *AUC* area under the curve, *CI* confidence interval, *e/h* events/hour, *NPV* negative predictive value, *ODI* oxygen desaturation index, *PPV* positive predictive value

### Predicting OSA

**Fig. 2:**
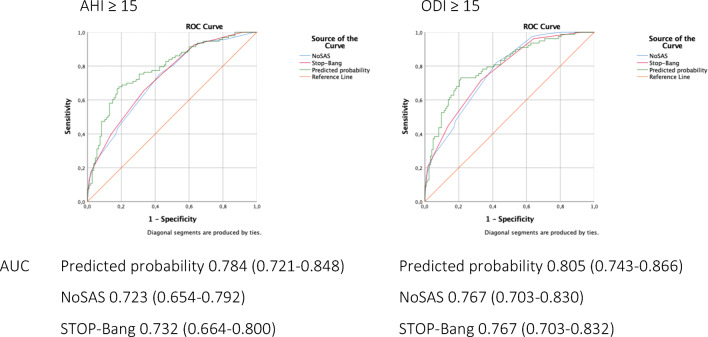
Discriminatory ability reported as area under the curve (AUC) (95% CI). The NoSAS score and the STOP-Bang questionnaire are presented as continuous variables. The green ROC curve shows the plotted predicted probability of gender, age, and BMI. The predicted probability performs similar to the NoSAS score and the STOP-Bang questionnaire (all comparisons with *p* value > 0.05). The ROC curves are presented at AHI ≥ 15 and ODI ≥ 15

Multivariate logistic regression analyses were performed in order to establish the association between various individual demographic and clinical variables and the presence and degree of OSA categorized by the AHI and the ODI. Gender, age, and BMI proved to be the strongest predictors for any OSA (AHI ≥ 5) (*p* < 0.001; *p* < 0.001; *p* = 0.004), moderate to severe OSA (AHI ≥ 15) (*p* < 0.001; *p* < 0.001; *p* < 0.001), ODI ≥ 5 (*p* = 0.001; *p* = 0.001; *p* = 0.001), and ODI ≥ 15 (*p* < 0.001; *p* < 0.001; *p* < 0.001). Gender, BMI, and self-reported history of hypertension proved to be or the strongest predictors for severe OSA (AHI ≥ 30) (*p* = 0.028; *p* < 0.001; *p* = 0.028) and ODI ≥ 30 (*p* = 0.024; *p* < 0.001; *p* = 0.034). The ROC curves of the estimated predictive probability, the NoSAS score, and the STOP-Bang questionnaire with cutoff points AHI ≥ 15 and ODI ≥ 15 are shown in Fig. 2. The AUC of the estimated predicted probability was 0.784 when differentiating for AHI ≥ 15 and 0.805 when differentiating for ODI ≥ 15. The predicted probability performs similar to the NoSAS score and the STOP-Bang questionnaire (all comparisons with *p* value > 0.05).

## Discussion

The present study shows that both the NoSAS score and the STOP-Bang questionnaire, but not the ESS, were equally useful to detect patients at high risk for OSA. In this cohort, the STOP-Bang questionnaire had the highest sensitivity, with a low specificity. The NoSAS score had a higher specificity and PPV, while maintaining a moderate to high sensitivity. The ESS had the highest specificity, with a low sensitivity. This is in correspondence with what was found by previous authors [8, 10, 11, 13, 18, 30]. The discriminatory ability of the NoSAS score and the STOP-Bang questionnaire was similar in relation to both the AHI and the ODI. However, due to the low specificity and positive predictive value of the STOP-Bang questionnaire, it is possible that the STOP-Bang will yield a large proportion of false-positive cases if used in a wrong patient group and therefore increase the number of unnecessary nocturnal recordings, whereas the NoSAS score describes higher specificity and positive predictive values, while maintaining a moderate to high sensitivity and negative predictive value.

The discriminatory ability of the NoSAS score and the STOP-Bang questionnaire as a categorical variable was compared with the discriminatory ability as a continuous variable. As expected, the discriminatory ability is higher when the instrument is used as a continuous variable. However, only for the STOP-Bang questionnaire, this difference proved to be significant. Previous studies have already suggested that the probability of moderate to severe OSA increases in direct proportion to the STOP-Bang score, and therefore, the questionnaire should be used as a continuous rather than as a categorical variable. Chung et al. suggested patients with a STOP-Bang score of 0 to 2 to be classified as being at low risk for moderate to severe OSA. Those with a STOP-Bang score of 5 to 8 can be classified as being at high risk for moderate to severe OSA. In patients with a STOP-Bang score of 3 or 4, specific combinations of positive items should be examined further to ensure proper classification [6]. The NoSAS score has previously been presented as categorical variable with various cutoff points [8, 10, 13, 14, 30]. However, according to our study results, a similar scoring system to the STOP-Bang questionnaire can be considered. Coutinho Costa et al. suggested a similar approach, prioritizing patients depending on their score. Patients with a score of 0–5 are to be classified as low probability of OSA—particularly moderate to severe OSA; a score ≥ 7 are to be classified as probable OSA; a score ≥ 12 as a high probability of OSA—particularly moderate to severe OSA [14].

In the present cohort, male gender, age, and BMI showed to be the strongest individual predictors for OSA severity based on the AHI and the ODI. The discriminatory ability of the three variables combined was similar to the discriminatory ability of the NoSAS score and the STOP-Bang questionnaire. In future, this might present interesting opportunities to design a screening tool based on only three variables. As an alternative, the weighing factor of the variables gender, age, and BMI could be set higher in the existing screening instruments. A similar approach was suggested by Chung et al. for the STOP-Bang questionnaire, introducing male gender, BMI, and neck circumference as high-risk variables [6].

### Clinical implications

This is the first study that evaluated the predictive performance of three different screening instruments with respect to both the AHI and the ODI. This is relevant, due to increasing evidence that the ODI has a higher reproducibility in the clinical setting [19–21]. Furthermore, significant differences in the severity of OSA have been described between patients with a similar AHI. Presumably, this is due to the fact that the morphology and duration of the apneas are not taken into account in the AHI [22]. In the present study, the NoSAS and STOP-Bang screening instruments both have a high discriminatory ability to predict OSA severity based on the AHI and the ODI. The ESS, however, was not able to detect patients at high risk for OSA and should, therefore, not be used as a screening instrument.

### Limitations and strengths

In general, the use of a retrospective analysis to validate the predictive value of different screening instruments is less ideal than a prospective study. In this observational study, however, our center had collected data prior to PSG monitoring, thus maintaining a high credibility for this retrospective study. Most patients were referred to the sleep clinic because they were suspected of having sleep-related problems. Therefore, it is possible that a selection bias was introduced, since the questionnaire was applied only to the suspected individuals. The great prevalence of OSA in this study population could affect the interpretation of the screening instruments. Contrarily, the present study has several important strengths: this is the first study that has evaluated the predictive value of different screening instruments on the ODI. As the ODI is gaining attention as new variable to classify OSA severity, this is an important new insight. Furthermore, all patients were evaluated with a full PSG and scored according to the current guidelines proposed by the American Academy of Sleep Medicine in 2012 [2].

## Electronic supplementary material

ESM 1 (DOCX 12 kb)

## Data Availability

The dataset is available on request from Christianne Veugen, Department of Otorhinolaryngology Head and Neck Surgery, Sint Antonius Hospital, Koekoekslaan 1, 3435 CM Nieuwegein, the Netherlands. E: c.veugen@antoniusziekenhuis.nl.

## Abbreviations

AHI: apnea-hypopnea index
AI: apnea index
ANOVA: analysis of variance
AUC: area under the curve
BMI: body mass index
CI: confidence interval
ESS: Epworth Sleepiness Scale
NC: neck circumference
NPV: negative predictive value
ODI: oxygen desaturation index
OSA: obstructive sleep apnea
PPV: positive predictive value
PSG: polysomnography
ROC: receiver operating characteristic
