# Supplementary material for: Prediction of obstructive sleep apnea: comparative performance of three screening instruments on the apnea-hypopnea index and the oxygen desaturation index
Source: Sleep Breath. 2020 Oct 24;25(3):1267–75. doi: 10.1007/s11325-020-02219-6 (PMC8376723; doi:10.1007/s11325-020-02219-6)
Supplement: Supplementary file 1 — (DOCX 12 kb) [file 11325_2020_2219_MOESM1_ESM.docx]

**Supplementary material**

*Stop-Bang score (cutoff ≥ 3 points)*

Snoring 1 point

Tiredness 1 point

Observed apneas 1 point

Pressure (Hypertension) 1 point

BMI > 35 1 point

Age > 50 1 point

Neck circumference > 40 cm 1 point

Sex: male 1 point

*NoSAS score (cutoff ≥ 8 points)*

Neck circumference > 40 cm 4 points

Obesity BMI 25-30 3 points

BMI ≥ 30 5 points

Snoring 2 points

Age > 55 4 points

Sex: male 2 points

*ESS score (cutoff ≥ 10 points)*

Sitting and reading 0 points 1 point 2 points 3 points

Watching TV 0 points 1 point 2 points 3 points

Sitting inactive in a public space 0 points 1 point 2 points 3 points

As a passenger in a car for 1 hour 0 points 1 point 2 points 3 points

Lying down to rest in the afternoon 0 points 1 point 2 points 3 points

Sitting and talking to someone 0 points 1 point 2 points 3 points

Sitting after lunch without alcohol 0 points 1 point 2 points 3 points

In a car, while stopped for traffic 0 points 1 point 2 points 3 points
